# Interspecies and intraspecies transmission of triple reassortant H3N2 influenza A viruses

**DOI:** 10.1186/1743-422X-4-129

**Published:** 2007-11-28

**Authors:** Hadi M Yassine, Mohammad Q Al-Natour, Chang-Won Lee, Yehya M Saif

**Affiliations:** 1 Food Animal Health Research Program, Ohio Agricultural Research and Development Center, The Ohio State University, Wooster OH, USA; 2 Department of Pathology and Animal Health, Faculty of Veterinary Medicine, Jordan University of Science and Technology, Irbid, Jordan

## Abstract

The triple reassortant H3N2 viruses were isolated for the first time from pigs in 1998 and are known to be endemic in swine and turkey populations in the United States. In 2004, we isolated two H3N2 triple reassortant viruses from two turkey breeder flocks in Ohio and Illinois. Infected hens showed no clinical signs, but experienced a complete cessation of egg production. In this study, we evaluated three triple reassortant H3N2 isolates of turkey origin and one isolate of swine origin for their transmission between swine and turkeys. Although all 4 viruses tested share high genetic similarity in all 8 genes, only the Ohio strain (A/turkey/Ohio/313053/04) was shown to transmit efficiently both ways between swine and turkeys. One isolate, A/turkey/North Carolina/03, was able to transmit from pigs to turkeys but not vice versa. Neither of the other two viruses transmitted either way. Sequence analysis of the HA1 gene of the Ohio strain showed one amino acid change (D to A) at residue 190 of the receptor binding domain upon transmission from turkeys to pigs. The Ohio virus was then tested for intraspecies transmission in three different avian species. The virus was shown to replicate and transmit among turkeys, replicate but does not transmit among chickens, and did not replicate in ducks. Identifying viruses with varying inter- and intra-species transmission potential should be useful for further studies on the molecular basis of interspecies transmission.

## 2. Introduction

Influenza A viruses are highly contagious pathogens that have been isolated form a wide variety of animals, including man, birds, swine, horses, minks, seals, whales, and most recently from cats and dogs [1-3]. Influenza A viruses are rarely known to cross species barriers [4,5], however, their interspecies transmission has always been a major concern. Although determinants of interspecies transmission are still not fully identified, many studies showed that the compatibility between the hemagglutinin (HA) protein of the virus and its corresponding receptor on the host cell is essential for establishing an infection in a specific host [6-8]. Pigs are known to be a major reservoir for H1N1 and H3N2 influenza viruses and have been hypothesized to act as intermediate host for interspecies transmission of influenza A viruses [6,9,10]. Turkeys on the other hand, are susceptible to a wide range of influenza A viruses and serve as an important host for these viruses [11,12]. Influenza infections in turkeys range from asymptomatic to severe disease, including respiratory tract disorder, depression, drop in eggs production and high mortality [11]. Between 1978 and 1981, our laboratory was the first to report on experimental and natural infections of turkeys with H1N1 swine influenza viruses [13,14].

In 1998, a new lineage of swine influenza viruses, triple reassortants (TR) H3N2, were isolated for the first time from pigs in the United States (U.S.) [15]. These viruses had genes derived from human (HA, NA, and PB1), Swine (NP, M, and NS) and avian viruses (PA and PB2) [16,17]. The H3N2 TR viruses are now endemic in swine populations in North America [17,18]. In 2003 and 2004, similar viruses (H3N2 TR) were isolated from turkeys in two different locations in the U.S. [19,20]. Later in the same year, we isolated another H3N2 TR virus from turkey breeder hens in Illinois that were vaccinated twice with a swine H3N2 TR virus. Infected turkeys experienced complete cessation of egg production, but had no other clinical signs. In a previous study (manuscript submitted) we observed major antigenic differences between turkey and swine H3N2 TR viruses. The antigenic relatedness (R-value) between the turkey viruses and the swine virus (vaccine strain) was less than 30% as expressed by the Archetti and Horsfall formula [21] based on hemagglutinin inhibition (HI) and virus neutralization (VN) tests. At least eight amino acid changes were observed at the antigenic sites of the HA1 molecule between the turkey viruses and the swine vaccine virus. Although the transmission of H3N2 TR viruses from pigs to turkeys was suggested in previous reports [19,20], no experimental work has been done to support this premise. Hence, we initiated this study to evaluate the interspecies transmission of these viruses between swine and turkeys, and to determine at the molecular level the basis for such transmission. Additionally, we tested one strain, A/turkey/Ohio/313053/04, that was shown to transmit between swine and turkeys for its intraspecies transmission in turkeys, chickens and ducks. Identifying viruses with different transmission potential between swine and turkeys will help in identifying the molecular determinants that control such transmission using the reverse genetics techniques.

## 3. Results

### 3.1 Interspecies transmission of H3N2 influenza viruses from pigs to turkeys

Three H3N2 influenza isolates of turkey origin and one H3N2 influenza isolate of swine origin were evaluated for their transmission from pigs to turkeys. Additionally, two H1N1 isolates of swine and turkey origins were included for comparison. All viruses were shown to replicate in pigs but with different efficiencies (Table 1). The A/turkey/Ohio/313053/04 and A/turkey/North Carolina/03 viruses replicated more efficiently than the other H3N2 viruses, with nasal swab titer of 2 × 10^6 ^and 2 × 10^6.6 ^50% tissue culture infectious dose (TCID_50_) per ml, respectively (Table 1). The H1N1 turkey strain, A/turkey/Ohio/88, showed the highest replication titer (2 × 10^8.1 ^TCID_50_/ml) among all viruses tested. The Ohio strain, A/turkey/Ohio/313053/04, elicited the highest antibody titer (1:360 HI) among all the H3N2 viruses tested (Table 1). Different patterns of transmission from pigs to turkeys were observed among the H3N2 TR viruses (Table 2). The H3N2 Ohio strain was transmitted from pigs to turkeys and virus was detected for more than two days in turkeys using the real-time reverse-transcription PCR (RRT-PCR). Four out of the eight contact turkeys got infected and three of them seroconverted to an average HI titer of 1:80. The A/turkey/North Carolina/03 virus was detected in three out of eight contact turkeys using the RRT-PCR, with one turkey detected positive for two days; however, none of the infected turkeys seroconverted. Viruses were successfully re-isolated using Madin-Darby Canine Kidney (MDCK) cells from the contact turkeys infected with A/turkey/Ohio/313053/04 and A/turkey/North Carolina/03 H3N2 viruses. On the other hand, although three out of eight and four out of eight swab samples were AIV-positive with RRT-PCR at two days post exposure (DPE) from turkeys in contact with pigs infected with A/turkey/Illinois/04 and A/swine/North Carolina/03, respectively, no viruses were isolated from any of the RRT-PCR positive samples and none of the turkeys seroconverted (Table 2). Both H1N1 viruses replicated in pigs, but none of them were detected in the contact turkeys as determined by RRT-PCR and HI tests.

**Table 1:** Interspecies transmission of H3N2 and H1N1 influenza viruses from pigs to turkeys; virus detection in inoculated pigs

**Virus**	No. positives 1 to 3 DPI*	**No. positives 4 to 6 DPI**	**No. positives for 2 or more days**	**Peak day of virus detection**	**Estimated average virus titer on peak day/ml**	**No. of animals seroconverted/total inoculated**	HI**** average titer at 14 DPI	**Virus isolation from swab samples**
**A/TK/IL/04 (H3N2)**	5/5**	3/3***	5/5	4DPI	2 × 10^4.5^	3/3	1:160	Positive
**A/TK/OH/04 (H3N2)**	5/5	4/4***	5/5	5DPI	2 × 10^6.0^	4/4	1:360	Positive
**A/TK/NC/03 (H3N2)**	5/5	4/4***	5/5	4DPI	2 × 10^6.6^	4/4	1:220	Positive
**A/SW/NC/03 (H3N2)**	5/5	3/3***	5/5	4DPI	2 × 10^4.7^	3/4	1:340	Positive
**A/TK/OH/88 (H1N1)**	5/5	4/4***	5/5	4DPI	2 × 10^8.1^	4/4	1:320	NT
**A/SW/OH/06 (H1N1)**	5/5	4/4***	5/5	4DPI	2 × 10^5.6^	4/4	1:160	NT

* Days post inoculation. Swabs were collected on daily bases and results are displayed in three days intervals.

** No. of pigs positive with RRT-PCR/No. of pigs inoculated.

*** Some pigs were euthanized at 3 DPI to collect organs and tissues for other studies.

**** Hemagglutinin inhibition.

NT Not Tested

**Table 2:** Interspecies transmission of H3N2 and H1N1 influenza viruses from pigs to turkeys; virus detection in turkeys in contact with inoculated pigs

**Virus**	No. positives 1 to 3 DPE*	**No. positives 4 to 6 DPE**	**No. positives 7 to 9 DPE**	**No. positives for 2 or more days**	**Peak day of virus detection**	**Estimated average virus titer on peak day/ml**	**No. of animals seroconverted/total exposed**	HI*** average titer at 14 DPE	**Virus isolation from swab samples**
**A/TK/IL/04 (H3N2)**	3/8**	0/8	0/8	0/8	2DPE	2 × 10^3^	0/8	-	Negative
**A/TK/OH/04 (H3N2)**	0/8	4/8	2/8	3/8	6DPE	2 × 10^3.12^	3/8	1:80	Positive
**A/TK/NC/03 (H3N2)**	0/8	2/8	1/8	1/8	5DPE	2 × 10^3.8^	0/8	-	Positive
**A/SW/NC/03 (H3N2)**	4/8	0/8	0/8	0/8	2DPE	2 × 10^3^	0/8	-	Negative
**A/TK/OH/88 (H1N1)**	0/8	0/8	0/8	-	-	-	-	-	NT
**A/SW/OH/06 (H1N1)**	0/8	0/8	0/8	-	-	-	-	-	NT

* Days post exposure. Swabs were collected on daily bases and results are displayed in three days intervals.

** No. of turkeys positive with RRT-PCR/total No. of contact turkeys.

*** Hemagglutinin inhibition.

NT Not Tested

### 3.2 Interspecies transmission of H3N2 influenza viruses from turkeys to pigs

We also evaluated the transmission of the H3N2 viruses from turkeys to pigs (Tables 3 and 4). As expected, all H3N2 viruses replicated in turkeys regardless of their isolation origin and were detected in the inoculated turkeys for at least six days, except for the A/swine/North Caroilina/03 virus that was detected for only four days. In general, the swab viral titers were lower than that from pigs, ranging from 2 × 10^2.8 ^to 2 × 10^3.3 ^TCID_50_/ml. Again, the Ohio and North Carolina turkey isolates replicated at the highest titers of 2 × 10^3.3 ^TCID_50_/ml. All viruses were shown to elicit antibody response in turkeys with the highest titer observed against the Ohio strain at 1:420 HI. Only the Ohio strain transmitted from the infected turkeys to the contact pigs as determined by RRT-PCR, HI test and virus isolation (Table 4). The first positive pig was detected at the 3 DPE, and the rest became positive at 5 DPE. All pigs infected with the Ohio strain seroconverted with an average HI titer of 1:320.

**Table 3:** Interspecies transmission of H3N2 influenza viruses from turkeys to pigs; virus detection in inoculated turkeys

**Virus**	No. positives 1 to 3 DPI*	**No. positives 4 to 6 DPI**	**No. positives 7 to 9 DPI**	**No. positives for 2 or more days**	**Peak day of virus detection**	**Estimated average virus titer on peak day/ml**	**No. of animals seroconverted/total inoculated**	HI*** average titer at 14DPI	**Virus isolation from swab samples**
**A/TK/IL/04 (H3N2)**	6/10**	7/10	NT	8/10	5DPI	2 × 10^2.9^	9/10	1:300	Positive
**A/TK/OH/04 (H3N2)**	7/10	5/10	2/10	8/10	4DPI	2 × 10^3.3^	4/6	1:420	Positive
**A/TK/NC/03 (H3N2)**	6/10	6/10	NT	6/10	4DPI	2 × 10^3.3^	3/10	1:80	Positive
**A/SW/NC/03 (H3N2)**	4/10	1/10	0/10	3/10	3DPI	2 × 10^2.8^	2/10	1:80	Positive

* Days post inoculation. Swabs were collected on daily bases and results are displayed in three days intervals.

** No. of turkeys positive with RRT-PCR/No. of inoculated turkeys.

*** Hemagglutinin inhibition.

NT Not Tested

**Table 4:** Interspecies transmission of H3N2 influenza viruses from turkeys to pigs; virus detection in pigs in contact with inoculated turkeys

**Virus**	No. positives 1 to 3 DPE*	**No. positives 4 to 6 DPE**	**No. positives 7 to 9 DPE**	**No. positives for 2 or more days**	**Peak day of virus detection**	**Estimated average virus titer on peak day/ml**	**No. of animals seroconverted/total exposed**	HI*** average titer at 14DPI	**Virus isolation from swab samples**
**A/TK/IL/04 (H3N2)**	0/5**	0/5	0/5	0/5	-	-	0/5	-	-
**A/TK/OH/04 (H3N2)**	1/5	5/5	5/5	5/5	5DPE	2 × 10^5^	5/5	1:320	Positive
**A/TK/NC/03 (H3N2)**	0/5	0/5	0/5	0/5	-	-	0/5	-	-
**A/SW/NC/03 (H3N2)**	0/5	0/5	0/5	0/5	-	-	0/5	-	-

* Days post exposure. Swabs were collected on daily bases and results are displayed in three days intervals.

** No. of pigs positive with RRT-PCR/total No. of contact pigs.

*** Hemagglutinin inhibition

### 3.3. Sequence analysis

**Figure 1:**
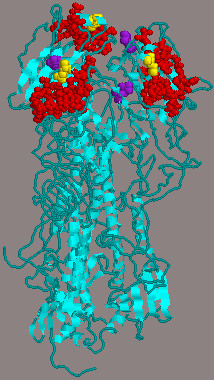
Cartoon representing the amino acid changes at the HA molecule of the A/turkey/Ohio/313053/04 H3N2 virus, that occurred upon replication and transmission of the virus between turkeys and pigs. Red: receptor binding domain (RBD). Yellow: the change at residue 190 that occurred upon transmission of the virus from turkeys to pigs. Violet: the change at residue 246 that occured in two of the inoculated pigs and one of the contact turkeys with inoculated pigs.

The two surface glycoproteins encoding genes, HA and NA, were amplified and sequenced from A/turkey/Ohio/313053/04 H3N2 virus isolated from directly inoculated pigs, pigs in contact with infected turkeys, directly inoculated turkeys and turkeys in contact with infected pigs. Pairwise sequence alignment showed two changes in the HA gene sequence upon replication and transmission of the virus from pigs and turkeys. The first change was observed at residue 190 (D to A) of the receptor binding domain (RBD) in viruses isolated from pigs in contact with infected turkeys (Figure 1). The other change was observed at residue 246 (S to N) in two of the inoculated pigs and one of the turkeys in contact with inoculated pigs (Figure 1). No changes were observed in the NA gene upon replication and transmission of the virus from pigs to turkeys and vise versa.

### 3.4. Intraspecies transmission of A/turkey/Ohio/313053/04 H3N2 virus in turkeys, chickens and ducks

To evaluate the transmission potential of H3N2 viruses in different avian species, we tested the intraspecies transmission of A/turkey/Ohio/313053/04 virus (strain that showed efficient transmission between pigs and turkeys) in turkeys, chickens and ducks (Table 5). The virus behaved differently in different avian species, where it was capable of replication in turkeys and chickens, but not in ducks. Although the replication titers in chickens were higher than those in turkeys, 2 × 10^6 ^and 2 × 10^3.4 ^TCID_50_/ml, respectively, no transmission was detected among chickens. The virus was detected for more than one day in 90% of the inoculated chickens, of which 62% seroconverted to an average titer of 1:216 HI. On the other hand, 80% of the inoculated turkeys were positive with RRT-PCR for influenza virus for more than two days, and all of them seroconverted to an average HI titer of 1:990. The very high HI average titer of the turkey serum was due to two turkeys that showed an HI titer of 5120 and 2560 respectively. Nine of the ten contact turkeys in the same cage became positive, two of which were positive at 3 DPE, while the rest were positive between 7 DPE and 9 DPE. Only two of the contact turkeys seroconverted to an HI titer of 1:120 HI units. The delay in infection in most of the contact turkeys would explain the negative HI tests (only two of the contact turkeys were positive) that were performed on serum samples collected at 14 day post exposure (DPE).

**Table 5:** Intraspecies transmission of A/TK/OH/313053/04 (H3N2) Influenza virus in chickens, ducks and turkeys

**Virus TK/OH/04 (H3N2)**	**No. positive 1 to 3 DPI/DPE**	**No. positive 4 to 6 DPI/DPE**	**No. positive 7 to 9 DPI/DPE**	**No. positive 10 to 12 DPI/DPE**	**Peak day of virus detection**	**Estimated average virus titer on peak day/mL**	**No. of animals seroconverted/total exposed**	**HI average titer**
**Infected chickens**	19/20	10/16*	NT	NT	2DPI	2 × 10^6^	10/16	1:216
**Contact chickens**	0/10	0/10	0/10	NT	-	-	0/10	-
**Infected ducks**	0/15	0/15	0/15	NT	-	-	0/15	-
**Contact ducks**	0/15	0/15	0/15	NT	-	-	0/15	-
**Infected turkeys**	13/15	8/10*	NT	NT	3DPI	2 × 10^3.4^	10/10	1:990
**Contact turkeys**	2/10	2/10	6/10	7/10 DPI	8DPE	2 × 10^3.5^	2/10	1:120

* Some of the inoculated turkeys and chickens were euthanized at 3DPI to collect tracheas for other studie

## 4. Discussion

Generally, influenza A viruses are considered host specific, nevertheless, some can overcome the species barrier and infect a new host. The mechanisms by which the influenza A viruses cross the species barriers and the molecular determinants that control such transmission are not well identified. Pigs have been hypothesized to play a role in interspecies transmission by acting as "mixing vessel" for the generation of reassortant viruses that might have the potential to jump from one species to another [22,23]. In 1998, a new lineage of swine viruses, H3N2 TR, emerged and caused influenza like illnesses in pig populations in the U.S. [15,16]. Similar viruses were later isolated from turkey breeder hens experiencing drop in eggs production and it was hypothesized that these viruses were transmitted from pigs to turkeys [19,20].

Our findings indicated the ability of certain H3N2 TR viruses to transmit between pigs and turkeys. Despite the high degree of molecular similarity between some of these viruses, like A/turkey/Illinois/04 and A/turkey/Ohio/313053/04 (>99% similarity in all genes), they behaved differently in the transmission experiments, with the A/turkey/Ohio/313053/04 transmitting both ways between the two species and the A/turkey/Illinois/04 virus not transmitting either way.

Regardless of the differences in transmission, all viruses were capable of replication in turkeys and pigs but to different titers. Furthermore, the A/turkey/Ohio/313053/04, the strain transmissible between pigs and turkeys, was shown to infect and transmit among turkeys, infect but did not transmit among chickens, and did not infect ducks.

We speculate that the H3N2 TR viruses, which have the HA gene from human lineage viruses, retain the receptor binding specificity to NeuAcα2,6Gal receptors similar to human influenza viruses. Val226 and Ser228 were expressed in the HA1 molecules of both turkey and swine triple reassortants, while Leu/Ile226 and Ser228 are usually expressed in the human viruses [24]. Leu, Ile, and Val are neutral non-polar amino acids, and substitutions between them most likely maintain the hydrophobic interactions and the proper conformation at the binding domain [25]. Gln226 and Gly228 are usually found in the HA1 molecules of avian viruses amino acids at these positions and are known to play a critical role in determining the receptor binding specificity [25]. Our unpublished work demonstrated the presence of substantial amount of NeuAcα2,6Gal receptors in turkey tracheas, which would explain the ability of these viruses to replicate in turkeys as well as in pigs that are known to express these receptors [6]. Although ducks were shown to express few NeuAcα2,6Gal receptors in their tracheas (unpublished work), the A/turkey/Ohio/313053/04 H3N2 virus was not able to replicate in ducks. The absence of a large number of NeuAcα2,6Gal receptors in ducks' tracheas may explain the inability of the A/turkey/Ohio/313053/04 H3N2 virus to replicate in ducks. However, there may be factors other than receptors distribution that contribute to host tropism of influenza viruses and more work is needed in this area.

While all viruses had the Asp (D) amino acid at residue 190 of the receptor binding domain (RBD), a D to A (Ala) change occurred upon the transmission of the A/turkey/Ohio/313053/04 virus from turkeys to pigs. The presence of either D (specific for SAα2,6- gal) or E (specific for SAα2,3- gal) at amino acid position 190 of the HA molecule in the H3 subtypes was reported in previous studies [26,27], however, our observation of (A) residue at this position is the first of its kind to our knowledge (sequencing was performed on the HA gene of the Ohio virus isolated from three different pigs in contact with infected turkeys). The role of (A) residue at position 190 in determining receptor binding specificity should be further investigated. In addition, the role of Asn (N) residue at position 246 of the HA molecule is not known and will be further studied in out laboratory.

Although all viruses were shed by pigs for more than 6 days, the A/turkey/Ohio/313053/04 and A/turkey/North Carolina/03 viruses replicated to higher titers than A/turkey/Illinois/04 and A/swine/North Carolina/03 viruses. This might be one of the possible reasons that allowed A/turkey/Ohio/313053/04 and A/turkey/North Carolina/03 viruses to transmit from pigs to turkeys (all animals were inoculated with the same virus titer). The A/turkey/Illinois/04 and A/swine/North Carolina/03 viruses were detected only on one day in contact turkeys by RRT-PCR, however, no viruses were obtained upon isolation attempts. The high sensitivity of the RRT-PCR might explain the ability to detect these viruses in contact turkeys, whereas the viruses were inefficient in replicating to a high titer in turkeys. In contrast, the A/turkey/Ohio/1988 H1N1 virus was shown to replicate to a very high titer in pigs (10^7.1 ^TCID_50_), but it did not transmit to turkeys. The above observations indicate the specificity of individual influenza A viruses, even within the same subtype (H3N2 TR in this case), in their ability to transmit between species.

Previous analysis of the A/swine/North Carolina/03 virus in our laboratory showed that it has a 13 amino acids stalk deletion in the NA protein (manuscript submitted). Shortened NA stalks might result in less efficient virus release, and hence lower virus titers [28,29]. This might explain our results from pigs and turkeys. However, the exact effect of NA stalk deletion is not clear because many chicken adapted H5, H7, and H9 viruses show different length stalk deletions and replicate to very high titer in poultry [30-32].

The identification of viruses with varying potential for interspecies transmission should be useful for reverse genetic studies to identify the gene(s) and the amino acid(s) residues that contribute to the transmission of these viruses between swine and turkeys. The use of the reverse genetics and site directed mutagenesis could also be helpful in deciphering the role of residues 190 and 246 of the HA molecule in receptor binding specificity and transmission of these viruses between swine and turkeys.

Interspecies transmission studies between swine (mammalian) and turkeys (avian) will enhance our understanding of the genetic factors that control transmission of influenza viruses and would help in improvement of surveillance strategies for early detection of influenza A viruses.

## 5. Materials and methods

### 5.1 Viruses

Four H3N2 TR viruses of turkey or swine origin were included in this study. Additionally, two H1N1 viruses (one turkey origin and one swine origin) were included for comparison. Two H3N2 turkey viruses, A/turkey/Illinois/04 and A/turkey/Ohio/313053/04, were isolated in MDCK cells in our laboratory in 2004, and were propagated in 9–10 days old embryonated chicken eggs (ECE) to make working stocks. One turkey virus, A/turkey/North Carolina/03 (H3N2) (passaged twice (P2) in MDCK cells), and one swine virus, A/swine/North Carolina/03 (H3N2) (unknown passage number), were kindly provided by Dr. Eric Gonder (Goldsboro Milling Co. Goldsboro, NC), and were propagated in 9–10 days old ECE to make working stocks. The turkey H1N1 (A/turkey/Ohio/1988) and swine H1N1 (A/swine/Ohio/06) viruses were isolated in ECE in our lab in 1988 and 2006, respectively. Both viruses were propagated once in ECE to make working stocks. The two H1N1 influenza viruses were included as controls for the transmission from pig to turkey, but not in the turkey to pig transmission study.

### 5.2 Virus isolation

Turkey tracheal swabs were used for inoculation of MDCK cell line maintained in Opti-MEM minimum essential medium (Invitrogen, Grand Island, NY) containing 0.5 μg/ml trypsin. The samples were passaged twice in MDCK cells and then used to inoculate 9–10 days old specific pathogen free (SPF) ECE to make working stocks.

### 5.3 Transmission studies

**Figure 2:**
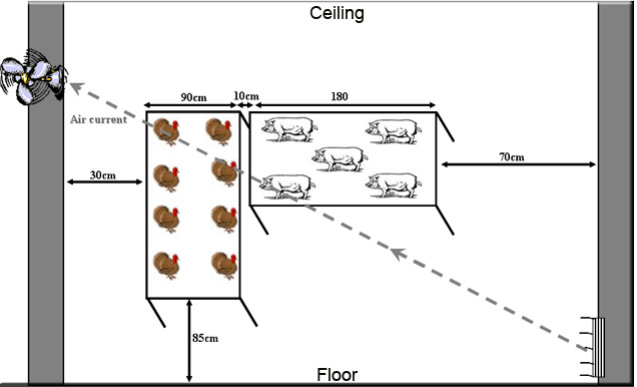
Schematic of the room used in the study of interspecies transmission of influenza viruses (swine to turkey transmission setting in this case). The air flowed from the experimentally infected animals to the contact uninfected animals. Scale is not proportional.

A schematic layout of the room used for the transmission studies is presented in Figure 2. The rooms were mechanically ventilated and the air was HEPA filtered at the intake and the exhaust. Briefly, the infected and contact animals were placed close to each other in two different cages (with rubber coated floors) to study the indirect transmission of H3N2 TR viruses between swine (large white breed) and specific pathogen free (SPF) turkeys. The direction of the air current was always from the infected animals' side to the contact animals' side. The animals received a virus titer of 10^7 ^TCID50 contained in 0.5 ml, and the contact animals were placed in the same room close to the infected animals at one day post inoculation (1 DPI). Nasal swabs from pigs and tracheal swabs from turkeys were collected on daily basis and were maintained in Brain Heart Infusion (BHI) media and were directly used for RNA extractions. Contact animals were always handled first.

Intraspecies transmission experiments with the Ohio virus (A/turkey/Ohio/313053/04) were performed in SPF turkeys, SPF chickens and commercial pekin ducks. The individual bird (n = 15 for turkeys and ducks, and n = 20 for chickens) received a virus titer of 10^7 ^TCID50 contained in 0.5 ml, and the contact animals (10 turkeys, 10 chickens and 15 ducks) animals were placed in the same cage at 1 DPI. Tracheal swabs were collected on daily basis and were maintained in BHI media and were directly used to do RNA extractions. Non-inoculated negative control animals were placed in a separate room and were treated like infected animals.

### 5.4 Antisera collection and HI test

Blood was collected from all animals at zero and fourteen days post inoculation/exposure (DPI/DPE) to test for antibodies to H3N2 and H1N1 influenza viruses. Sera were harvested and inactivated at 56°C for 30 min before being used in hemagglutinin inhibition (HI) test. The HI test was carried out as previously described [33]. Titers were determined by using twofold serial dilutions of antisera (25 μl), 4 HA/25 μl units of homologous antigen and a 0.5% suspension of turkey erythrocyte per test well.

### 5.5 RNA extraction and real-time RT-PCR

RNA extraction and RRT-PCR reactions were performed as previously described [34-36]. Briefly, swab samples in 1.5 ml BHI media were vortexed for 5 seconds then left standing for 15 min to precipitate the debris. Of the 1.5 ml swab sample, 300 μl were used for RNA extraction using the RNeasy kit (Qiagen, Valencia, CA). RRT-PCR was performed in 25 μl reaction volume using the Qiagen one-step RT-PCR kit with the following conditions: 10 pmol of each primer, 320 μM each dNTP, 0.12 μM FAM labeled probe, 13 units RNase inhibitor, 1 μl enzyme mix, 8 μl of RNA sample, and water was added to get a total volume of 25 μl. The RRT-PCR conditions were: 50°C for 30 min, 95°C for 15 min, and 45 cycles of 1 sec at 94°C and 20 sec at 60°C. Reactions were run in the Cephid Smartcycler thermocycler (Utech Products, Inc.; Schenectady, NY 12305).

### 5.6 Standard curve for virus titer estimation

To estimate the virus titer in the infected animals, we established a standard curve based on one turkey and one swine H3N2 viruses of known TCID_50 _titer. Briefly, RNA was extracted from A/turkey/Illinois/04 and A/swine/North Carolina/03 and serial dilutions were prepared. The serially diluted RNA was used to run the RRT-PCR as described above and a standard curve was established.

### 5.7 Sequence analysis and molecular graphic visualization

The HA1 and NA genes of the A/turkey/Ohio/313053/04 virus were amplified from viruses obtained from directly inoculated pigs, pigs in contact with infected turkeys, directly inoculated turkeys and turkeys in contact with infected pigs. Both genes were amplified with standard reverse transcription (RT) PCR using influenza specific primers and the one-step RT-PCR kit (Qiagen) following the manufacturer instructions. The RT-PCR products were separated by electrophoresis on 1% agarose gel, and amplicons of the right size were excised from the gel and purified with Qiaquick gel extraction kit (Qiagen). Sequencing was done at the Ohio Agricultural Research and Development Center (OARDC) sequencing facility using the ABI Prism 3100 automated sequencing machine (Applied Biosystems, Foster City, CA 94404). Pairwise sequence alignments were performed in the MegAlign program (DNASTAR, Madison, Wis.) to determine nucleotides and amino acids sequences similarity. Amino acid changes in the HA protein of different isolates were located using the Rasmol software (v2.6.4) (Biomolecular Structures Group, Hertfordshire, UK) on the HA structure of H3 subtype influenza virus, A/Aichi/2/68, (1HGG) downloaded from the Protein Data Bank website [37,38].
